# Evaluation of quality of life and self-efficacy in adolescents with amblyopia

**DOI:** 10.25122/jml-2020-0035

**Published:** 2022-04

**Authors:** Akram Mazlominezhad, Farhad Adhami Moghadam

**Affiliations:** 1. Department of Nursing and Midwifery, Tehran Medical Sciences, Islamic Azad University, Tehran, Iran; 2. Department of Ophthalmology, Tehran Medical Sciences Branch, Islamic Azad University, Tehran, Iran

**Keywords:** amblyopia, self-efficacy, QoL

## Abstract

Amblyopia is an acquired defect due to a lack of visual stimulation. Self-efficacy is one of the factors affecting the quality of life (QoL) of individuals. Low self-efficacy can decrease cognitive and behavioral functioning, whereas increased self-efficacy leads to a change in treatment acceptance behavior and, subsequently, physical and mental health. This study aimed to determine the QoL and self-efficacy in adolescents with amblyopia. This descriptive cross-sectional study was performed on 300 patients referred to hospitals affiliated with Mashhad University of Medical Sciences, Iran, between 2015 and 2016. Data collection tools included: 1) demographic questionnaire, 2) general self-efficacy scale, and 3) WHO QoL questionnaire. Data were analyzed using SPSS software 24 using descriptive statistics. The results showed that the mean percentage in QoL was 45.43% (weak), and the mean score in self-efficacy was 21.66%, with a standard deviation of 8.10. There was also a positive and significant relationship between patients' self-efficacy and QoL on each dimension. This study showed that demographic characteristics had no significant relationship with any of the variables of QoL and self-efficacy. However, data analysis showed a significant and positive relationship between self-efficacy and QoL.

## Introduction

Amblyopia is a visual system disorder characterized by visual impairment in the eye but physically normal and without any structural abnormalities [1]. It is seen in people with monocular strabismus, anisometropia and isoametropia, and visual impairment in general [2]. The prevalence of amblyopia is challenging to assess and varies in the literature. Amblyopia varies from 1.5% in healthy children and 3.5–4.5% in children with visual impairments. Furthermore, most statistics indicate that about 2% of the population develops amblyopia [3]. The prevalence of amblyopia globally is two to five percent, with an estimated prevalence of 4.1% in Iran [4]. The most critical time for children is from birth to 2 years.

Quality of vision significantly impacts people's quality of life (QoL) and performance and increases individual and government costs [5]. Therefore, the dimensions of QoL are considered key elements in public policymaking and are referred to as indicators of social development. Characteristics of this structure, such as dynamics, multidimensionality, and various assumptions, make it widely used in health studies. Undoubtedly, such attention has increased the volume of studies and investigation of QoL [6]. The expansion of industrialization and advances in technology focusing on the quantitative dimension of human life and the neglect of qualitative aspects over the past few decades has come to the attention of scholars focused on improving living conditions and the QoL of human beings [6]. 

Self-efficacy can affect many dimensions of an individual [7]. Low self-efficacy can decrease cognitive and behavioral functioning, whereas increased self-efficacy leads to a change in treatment acceptance behavior and, subsequently, physical and mental health [8]. Self-efficacy, as an effective factor in improving QoL, emphasizes one's understanding of his or her skills and abilities to perform successfully. In other words, self-efficacy affects perceptions of adaptive behavior and the choice of environment and conditions individuals strive to achieve [9]. Low self-efficacy makes people feel helpless and unable to control their life events [10]. Furthermore, self-efficacy beliefs influence individuals' patterns of thought and emotional actions. 

Identifying the factors that contribute to this dilemma will play a significant role in reducing the financial and psychological costs of vision impairment. Physicians should be aware of the prevention and treatment of amblyopia, particularly the factors that contribute to savings costs. Children should also be mindful of vision impairments, strabismus, and other causes that can help them adjust to this defect. Therefore, this study aimed to determine QoL and self-efficacy in adolescents with amblyopia to identify necessary strategies.

## Material and methods

This descriptive cross-sectional study included 300 individuals referred to the specialized clinic of Khatam al-Nabia and Farabi hospitals in Mashhad, Iran, between 2015 and 2016. Inclusion criteria were ages 11 to 18 years and clinically diagnosed amblyopia with 2 lines of vision on the visual acuity chart (Jarret Snellen). 

The data collection tools included a demographic questionnaire and a general self-efficacy scale. The scale had 20 items with two general and social self-efficacy subscales reduced to a 10-item scale in 1981. Questions are scored on a 4-point Likert scale. Thus, on a 10-point scale ranging from 40-to 40, the score will be 20–20 low self-efficacy, 21–30 moderate self-efficacy, and higher than 30 high self-efficacy [11]. In the Rajabi study, the concurrent validity coefficient and the Rosenberg self-esteem scale were significant. This questionnaire applies to the Iranian population [12]. 

Finally, the WHOQOL-BREF 26-item QoL Questionnaire was used. The 26-item QoL Questionnaire is based on the Likert scale of never-ever-adjusted domains. The scale is scored on a scale of 1 to 5 points. The maximum score a person can get from this scale is a maximum of 100 and a minimum of 0. These sub-scales are physical health (7 questions), mental health (6 questions), social relationships (3 questions), environmental health (8 questions), and an overall score (2 questions). Initially, a raw score is obtained for each subscale, which must be converted to a standard score of 0 to 100% by a formula. A higher score indicates a higher QoL. After the scores are converted, QoL is classified into three levels: high (75 and above), moderate (50–74), and low (below 50) [13]. In a study of questionnaire validation in foreign countries, overall intra-group correlation coefficients were above 0.7 [14]. The reliability of the test with the subscales was as follows: physical health 0.77, mental health 0.77, social relations 0.75, environmental health 0.84, and 0.82 for the whole questionnaire.

### Statistical analysis

This study used descriptive statistics and correlation analyses to evaluate the QoL self-efficacy in adolescents with amblyopia. All analyses were done using SPSS 24 software, and also the significance level was considered 0.05 with a confidence interval of 85%.

## Results

The demographic characteristics of participants are presented in Table 1. The average percentage of QoL was 45.43%, and most people had poor QoL (Table 2). The mean score of self-efficacy for patients was 21.63±8.10. Also, the highest score among the patients was 38, and the lowest score was 12, with the maximum score being 40 and the minimum score 10. 175 (58.3%) individuals had low self-efficacy, 55 (18.3%) had moderate self-efficacy, and 70 (23.3%) had high self-efficacy. The results showed that none of the demographic characteristics were correlated with the QoL score (Table 3, P>0.05). None of the demographic characteristics were correlated with self-efficacy scores (Table 4, P>0.05). The results also showed a positive and significant relationship between self-efficacy and QoL in patients (P<0.01, r=0.550). The higher the self-efficacy, the higher the QoL, and *vice versa*.

**Table 1.** Frequency and percentage of demographic characteristics among adolescents with amblyopia.

**Items**	**Subgroups**	**Rate (%)**	**Total No.**
**Age group**	11–12 y	22.7	68
	13–14 y	10.3	31
	15–16 y	31.0	93
	17–18 y	36.00	108
**Sex**	Male	7.47	143
	Female	3.52	157
**History of amblyopia in the family**	+	3.79	238
	-	7.20	62
**Patient status at birth**	Premature	62.0	186
	Not premature	38.0	114
**NICU admission status**	Admitted	7.85	257
	Not admitted	3.14	43

**Table 2.** QoL dimensions in adolescents with amblyopia.

	**Poor** **level**	**Moderate** **level**	**High** **level**
**QoL**	163 54.3%	491 6.3%	88 29.3%
**Physical health**	196 65.3%	78 26.0%	26 8.7%
**Mental health**	203 67.7%	71 23.7%	26 8/7%
**Environmental health **	198 66.0%	77 25.7%	25 8/3%
**Social communication **	169 56.3%	103 34.3%	28 9.3%
**Public health **	214 71.3%	63 21.0%	23 7.7%

**Table 3.** Relationship between some demographic characteristics, demographic factors, and QoL, P>0.05.

**QoL level**		**Poor level**	**Moderate level**	**High level**	**Total**
**Sex**	Male	78	23	42	143
	Female	85	26	46	157
**Father's education**	Illiterate	143	45	76	264
Under diploma	14	4	10	28
Above diploma	6	0	2	8
**Mother's education**	Illiterate	129	33	64	226
Under diploma	27	10	16	53
Above diploma	7	6	8	21
**Family history of illness**	+	13	39	68	238
	-	32	10	20	62
Born premature	+	98	25	63	186
	-	65	24	25	114
**Admission in NICU**	+	136	40	81	257
	-	27	9	7	43

**Table 4.** Relationship between some demographic characteristics, individual factors, and family with self-efficacy, P>0.05.

**Self-efficacy level**		**Poor level**	**Moderate level**	**High level**	**Total**
**Sex**	Male	84	24	35	143
	Female	91	31	35	157
**Father's education**	Illiterate	152	52	60	264
	Under diploma	16	3	9	28
	Above diploma	7	0	1	8
**Mother's education**	Illiterate	130	45	51	226
	Under diploma	34	6	13	53
	Above diploma	11	4	6	21
**Family history of illness**	+	138	43	57	238
	-	37	12	13	62
**Born premature**	+	106	33	47	186
	-	69	22	23	114
**Admission in NICU**	+	145	49	63	257
	-	30	6	7	43

## Discussion

According to the results, the average QoL was 45.43%, which is poor. In a study conducted in Turkey the mean score was 66.4% [15], 51% in Japan [16], 65% Brazil [17], and 83% in France [18]. In a study by Azizi *et al.*, who examined the QoL of patients with pigmented retinitis in Shiraz, researchers concluded that retinitis pigmentosa and decreased visual acuity could significantly decrease patients' QoL [19]. Also, in a study on chronic eye diseases (diabetic retinopathy, aging-induced degeneration, glaucoma, and cataract), patients with a lower vision score of 20/70 had less than half of the QoL score [20]. In this study, the QoL of patients was lower than in other countries, indicating the need for further studies to investigate the causes of differences. Tou *et al.* conducted a study on the impact of eye strabismus or strabismus on QoL of Han Chinese adolescents in 2016. The results showed that QoL in Han Chinese adolescents with strabismus compared to those without strabismus had lower scores [21]. These differences can be due to the severity of the disease – the lower the vision, the lower the QoL. However, we should consider that some other factors can affect the QoL. Rafii, for example, stated that the high QoL of most of the patients studied could be attributed to their moderate and good economic status and the persistence of ostomy in most of them [22]. It is important to be aware of the QoL in the health care system today. 

Also, the validity of the QoL questionnaire was evaluated by numerous studies, and it was suggested that the QoL questionnaire could be used domestically [23]. Cronbach's alpha coefficients for this questionnaire were reported in different countries: in Canada 0.88 [24], in Costa Rica 0.81 [25], in Germany 0.81 [26], in France 0.82 [27], in Spain it was 0.84 [28.]The reliability of this scale in the Rabbani Conscientious Research (1991) was measured by Cronbach's alpha test and Cronbach's alpha coefficient of 0.8 [29]. Also, this questionnaire was tested for its reliability with Cronbach's alpha of 0.8, previously [23]. It is very important to measure QoL when performing QoL interventions. Most of these relate to patients with chronic diseases whose definitive treatment is unknown [30]. Fassino states that awareness of QoL is an essential indicator of QoL today, and since QoL encompasses many aspects such as physiological, functional, and individual aspects, it is important to evaluate.

Proper QoL needs to be taken into account [31]. Measuring QoL in clinical studies also leads to a closer relationship between the client and the care team [30]. Therefore, according to the results of these studies, a strategy should be planned to increase QoL among these patients. Work out strategies such as counseling classes and training sessions for patients to enhance their QoL. The mean score of self-efficacy for patients was 21.66, with a standard deviation of 8.10. In another study by Rafii *et al.* (2012), the association between self-efficacy and QoL was relatively high [22]. The results of the present study are in line with the study of Wu *et al.* [32]. The high self-efficacy of the studied patients can be attributed to the persistence of ostomy in most of the studied units. There was a significant relationship between the persistence of ostomy and patients' self-efficacy. Bandura considers perceived self-efficacy as an important predictor of intention and ability to accept health patterns and believes that self-efficacy can be achieved by creating a suitable context for acquiring the skills and knowledge needed to succeed [33]. A person with low self-efficacy is less likely to attempt new health behaviors or change the behavior they are used to [34]. 

Therefore, as the majority of people in this study had low self-efficacy, it is expected that some measures will be taken to increase their self-efficacy. The results showed that none of the demographic characteristics were related to QoL and self-efficacy. In a study examining the impact of adaptive teaching on agricultural students' focused on weather and climatology, there was a significant relationship between age, marital status, and QoL. The study results showed that women had a higher mean age than men. Furthermore, there were more unmarried women than men, and as it was stated, people of higher age and unmarried had lower QoL [35]. Other studies also showed that age had a significant relationship with postoperative self-efficacy, with age decreasing self-efficacy and younger individuals having better self-efficacy [36]. Nevertheless, in some studies, there was no significant relationship [37]. The findings of Rafii *et al.* (2012) also showed that self-efficacy and QoL in osteomyelitis were lower in unmarried people than in married people. Moreover, it showed that self-efficacy was lower in unmarried people than in married people [22]. However, in the study of Wu *et al.*, there was no significant relationship between self-efficacy and marital status [32].

The results showed a positive and significant relationship between the self-efficacy of patients with QoL and each of its dimensions. This result is in line with other studies. It is concluded that patients with a positive attitude, de-stressing ability, and decision-making ability about their illness and who believe in their abilities as a whole have high QoL. A previous study showed a significant relationship between QoL and general health variables, social support, and self-efficacy [38]. Another study found self-efficacy correlated with QoL, disease recovery, the severity of illness, and psychological adjustment [39]. Overall, personal efficacy affects the physical health of individuals. Some studies found that people who believed they could relieve their pain could do so. Researchers have argued that coping strategies that increase personal efficacy can greatly increase the production of body endorphins (natural painkillers). Personal self-efficacy also helps improve physical illness and facilitates many health-promoting behaviors (such as exercise, weight control etc) in the individual [22].

The findings also showed that self-efficacy is most directly correlated with the social dimension of QoL. This finding is in line with the results of Kohno *et al.* among gastric cancer patients [40]. In general, people's beliefs about their efficiency play an important role in organizing, creating, and managing events that affect their lives, including social events. Therefore, a strong sense of self-efficacy is associated with great social success and is referred to as a key concept in social psychology [41]. Knowledge of the QoL in the healthcare system is important today. The importance of QoL assessment is to the extent that some call it the most important goal of QoL interventions. Most of this relates to patients with chronic diseases whose definitive treatment is not known [30]. Fassino states that awareness of QoL is an essential indicator of QoL today, and since QoL encompasses many aspects such as physiological, functional, and individual aspects, it is important to evaluate. Correct QoL needs to be taken into account [31]. Measuring QoL in clinical studies also leads to a closer relationship between the client and the care team [30].

Considering the concepts of QoL and self-efficacy as important concepts is essential for determining the effect of care on patients [42]. Self-efficacy is directly related to healthy behaviors and indirectly affects healthy behaviors toward achieving goals. Self-efficacy affects the amount of endurance, commitment, and effort to achieve a goal, and how well we have met our behavioral criteria determines our sense of self-efficacy [38]. People with high self-efficacy who are confident in their abilities actively participate in health promotion programs [22]. Obviously, participating in health programs increases people's QoL and affects their overall self-efficacy, perception of adaptive performance and behaviors, and the environment and conditions that individuals strive to achieve [9]. Accordingly, those who are more confident in their abilities and have higher self-efficacy are more effective in acquiring and applying skills, thus, establishing successful social interactions and resolving individual conflicts - improving their QoL. Therefore, high self-efficacy improves one's QoL and increases hope and motivation [43]. According to researchers, low self-efficacy is associated with low self-esteem, pessimistic self-esteem, and self-efficacy. People with low self-efficacy avoid any action they believe is beyond their ability. On the other hand, a strong sense of self-efficacy enhances performance and well-being [44]. Studies also showed that low self-efficacy is characterized by emotion-focused coping strategies and symptoms of anxiety and depression, depression, psychosomatic symptoms, and negative well-being. So a sense of self-efficacy can affect all aspects of life. Future interventions are needed to increase the QoL by holding educational classes and considering programs to increase self-efficacy.

## Conclusion

This study showed that demographic characteristics had no significant relationship with any of the variables of QoL and self-efficacy. However, data analysis showed a significant and positive relationship between self-efficacy and QoL. 

## Acknowledgments

### Conflict of Interests

The authors declare no conflict of interest.

### Consent to participate

Written informed consent was obtained from the participants.

### Data availability

Further data is available from the corresponding author on reasonable request.

### Personal thanks

The authors would like to thank the patients participating in the study.

### Authorship

FAM contributed to conceptualizing the study, methodology, and editing the manuscript. AM contributed to data collection, data analysis, and writing the original draft.

## References

[1] Shui HL (2016). Instant clinical Diagnosis in Ophthalmology Pediatric Ophtalmology..

[2] Bokhary KA (2016). Vision-related Quality of Life in Children with Amblyopia.. Optom open access..

[3] DM E, Jane C. (2016). The relationship between emotional intelligence, communication skills and stress among Iranian premier league referees.. Basic Clin Sci CoursePediatric Ophthalmol StabismusAmerican Acad Ophthalmol Last Major Revis 2015-2016.

[4] Levi DM (2013). Linking assumptions in amblyopia.. Vis Neurosci..

[5] Li J, Thompson B, Deng D, Chan LY (2013). Dichoptic training enables the adult amblyopic brain to learn.. Curr Biol..

[6] Gholamreza G, Alireza K, Hamzeh N (2012). Trend Study Of Quality Of Life In Iran..

[7] Madadkar S, Basiri M (2018). Effect of Relaxation Jacobson on QoL and self-efficacy in Patients Undergoing Hemodialysis.. Complement Med J Fac Nurs Midwifery.

[8] Baghaie Lake M, Rahimi S, Adib M, Kazem Nejad Leili E, Monfared A (2014). redictive Personal factors of quality of life in hemodialysis patient.. Journal of Holistic Nursing And Midwifery..

[9] Iskender M (2009). The relationship between self-compassion, self-efficacy, and control belief about learning in Turkish university students.. Social Behavior and Personality..

[10] Schultz DP, Schultz SE (2016). Theories of personality..

[11] Amirpour B, Shahbazirad A (2018). Investigating confirmatory Factor Analysis, reliability, and validity of the social self-efficacy scale for college students.. Quarterly of Educational Measurement..

[12] Rajabi GH (2006). Survey the Validity and Reliability of the General Self Efficacy in Students of Psychology and Educational Science in Faculty of Ahvaz University and Azad University of Marvdasht.. J Mod Educ Thought.

[13] Chavoshian SA, Moeini B, Bashirian S, Feradmal J (2015). The role of spiritual health and social support in predicting nurses' QoL.. J Educ Community Heal.

[14] Fayers PM, Machin D (2013). Quality of Life: The Assessment, Analysis and Interpretation of Patient-reported Outcomes, Second Edition..

[15] Toprak AB, Eser E, Guler C, Baser FE, Mayali H (2005). Cross-validation of the Turkish version of the 25-item National Eye Institute Visual Functioning Questionnaire (NEI-VFQ 25).. Ophthalmic Epidemiol..

[16] Suzukamo Y, Oshika T, Yuzawa M, Tokuda Y (2005). Psychometric properties of the 25-item National Eye Institute Visual Function Questionnaire (NEI VFQ-25), Japanese version.. Health Qual Life Outcomes..

[17] Labiris G, Katsanos A, Fanariotis M, Tsirouki T (2008). Psychometric properties of the Greek version of the NEI-VFQ 25.. BMC Ophthalmol..

[18] Simão LM, Lana-Peixoto MA, Araújo CR, Moreira MA, Teixeira AL (2008). The Brazilian version of the 25-Item National Eye Institute Visual Function Questionnaire: translation, reliability and validity.. Arq Bras Oftalmol..

[19] Fatemeh A, Mohammad AA, Abbas R, Hossein N, Mehdi TS (2018). Quality Of Life In Patients With Retinitis Pigmentosa In Shiraz..

[20] Asgari S, Nedjat S, Hashemi H, Shahnazi A, Fotouhi A (2012). Quality of Life in the Group of Patients with Chronic Eye Disease.. Iranian Journal of Epidemiology..

[21] Ahmadi F, Salar A, Faghihzadeh S (2004). Quality of life in Zahedan elderly population.. Hayat..

[22] Rafii F, Naseh L, Yadegary MA (2012). Relationship between Self-efficacy and QoL in Ostomates.. Iran J Nurs.

[23] Karimi Z, Andam R (2015). Investigation of Procrastination at Work and and its its Relationship with Self-efficacy and Dimension of Perfectionism (Case Study: Sport and Youth Office in Province of Hamedan).. Journal of Human Resource Management in Sport..

[24] Osei WD, Rasali DP, Hawkey JB, McCrae SM, Johnson SJ (2007). Cost Implications of Falls Injury Hospitalizations in Older Residents in Saskatchewan, Canada.. Annals of Epidemiology..

[25] Molla Jafar H, Salabifard S, Mousavi SM, Sobhani Z (2015). The Effectiveness of Group Training of CBT-Based Stress Management on Anxiety, Psychological Hardiness and General Self-Efficacy Among University Students.. Glob J Health Sci..

[26] Koch R, Wittekindt C, Altendorf-Hofmann A, Singer S, Guntinas-Lichius O (2015). Employment pathways and work-related issues in head and neck cancer survivors.. Head Neck..

[27] Bolaños-Medina A (2014). Self-efficacy in translation.. Transl Interpret Stud J Am Transl Interpret Stud Assoc.

[28] Razaviyayn M, Padash Z, Moradi A (2012). The determination of self-esteem, self-efficacy and achievement motivation measures in predicting women's QoL.. Interdiscip J Contemp Res Bus.

[29] Amirpour B, Shahbazirad A (2018). Investigating confirmatory Factor Analysis, reliability, and validity of the social self-efficacy scale for college students.. Quarterly of Educational Measurement..

[30] Ahmadi F, Salar A, Faghihzadeh S (2004). Quality of life in Zahedan elderly population.. Hayat..

[31] Fassino S, Leombruni P, Abbate Daga G (2002). Quality of life in dependent older adults living at home.. Arch Gerontol Geriatr..

[32] Wu HK, Chau JP, Twinn S (2007). Self-efficacy and quality of life among stoma patients in Hong Kong.. Cancer Nurs..

[33] Denoyelles A, Hornik S, Dixon K, Johnson R (2014). Exploring the Dimensions of Self-Efficacy in Virtual World Learning: Environment, Task, and Content.. Learning and Teaching..

[34] Ding J, Levi DM (2011). Recovery of stereopsis through perceptual learning in human adults with abnormal binocular vision.. Proc Natl Acad Sci U S A..

[35] Shafiei Z, Babaee S, Nazari A (2014). Relationship Between Self Efficacy And Quality Of Life In Coronary Artery Bypass Graft Surgery In Isfahan Chamran Hospital, 2010-2011..

[36] Khoshnazar T, Farmanbar R, Moghamnia M, Izadi Tameh A (2014). Relevance self-efficacy with anxiety and depression among patients receiving hemodialysis referred to hemodialysis unit at educational-therapeutic center in Rasht.. J Urmia Nurs Midwifery Fac.

[37] Smaeli M, Alikhani M, Hosseini F (2005). The QoL and self-efficacy of the patients under hemodialysis.. Iran J Nurs.

[38] Behnam Moghadam M, Behnam Moghadam A, Yarian S, Hosseini SM, Mohammad Hosseini S (2014). Predicting the Quality of Life Based on Public Health, Social Support and Self Efficacy in Cardiovascular Patients.. Armaghane Danesh..

[39] Chih AH, Jan CF, Shu SG, Lue BH (2010). Self-efficacy affects blood sugar control among adolescents with type I diabetes mellitus.. J Formos Med Assoc..

[40] Kohno Y, Maruyama M, Matsuoka Y, Matsushita T (2010). Relationship of psychological characteristics and self-efficacy in gastrointestinal cancer survivors.. Psychooncology..

[41] Morowatisharifabad M, Rouhani Tonekaboni N (2008). Perceived self-efficacy in self-care behaviors among diabetic patients referring to Yazd Diabetes Research Center.. Journal of Birjand University of Medical Sciences..

[42] Canam C, Acorn S (1999). Quality of life for family caregivers of people with chronic health problems.. Rehabil Nurs..

[43] Robinson-Smith G (2002). Self-efficacy and QoL after stroke.. J Neurosci Nurs.

[44] Shojaei F (2008). Quality of Life in Patients with Heart Failure.. Hayat..

